# Stepwise dose reduction and discontinuation of bDMARD in rheumatoid arthritis: a prospective cohort study of flare-free population, flares, and predictive markers

**DOI:** 10.1186/s13075-025-03672-y

**Published:** 2025-11-04

**Authors:** Yuji Nozaki, Kazuya Kishimoto, Daisuke Tomita, Tetsu Itami, Chisato Ashida, Toshihiko Shiga, Hirotaka Yamazawa, Kaori Ishimura, Yumi Morimoto, Rika Fukuda, Koji Kinoshita

**Affiliations:** Department of Hematology and Rheumatology, Kindai University Faculty of Medicine, Osaka-sayama, 589-8511 Osaka Japan

**Keywords:** Rheumatoid arthritis, Biological DMARDs, Dose flare prediction, Ultrasound

## Abstract

**Background:**

This study investigated the clinical outcomes of stepwise dose-reduction and discontinuation protocols for biological disease-modifying anti-rheumatic drugs (bDMARD) in patients with stable rheumatoid arthritis (RA), and explored predictors of disease flares.

**Methods:**

Seventy-one patients in clinical remission for ≥ 6 months were enrolled in the Reduction group. In Phase 1, dosing intervals were extended to 1.5 and then 2.0 times the standard schedule for patients who maintained low disease activity (LDA) for ≥ 12 months. Patients who sustained LDA (*n* = 41) advanced to Phase 2 for complete bDMARD discontinuation. Seventy-one matched patients who continued standard therapy served as controls.

**Results:**

In Phase 1, 12-month flare-free population rates were 80.6% in the Reduction group and 87.1% in the control group. Adverse events leading to discontinuation were rare in both groups. Gray-scale ultrasound findings were associated with flares during dose reduction (Wald χ²=2.3, *p* = 0.04, OR 1.06). In Phase 2, 47.5% of patients experienced flares after bDMARD discontinuation, and the 24-month flare-free population rate was significantly lower in the Reduction group (52.5%) compared to controls (86.6%) (HR 4.6, 95% CI: 2.2–11.0, *p* < 0.001). Serious adverse events were infrequent and occurred only in the control group. Power Doppler scores and ACPA-positivity were predictive of flares, while concomitant methotrexate use reduced flare risk.

**Conclusions:**

Tapering bDMARD may maintain disease control short-term, but discontinuation increases flare risk. Ultrasound findings and ACPA-positivity are valuable predictors, and concomitant methotrexate use may help prevent flares after discontinuation.

**Supplementary Information:**

The online version contains supplementary material available at 10.1186/s13075-025-03672-y.

## Introduction

Rheumatoid arthritis (RA), a chronic autoimmune disease characterized by persistent joint inflammation and systemic complications, imposes significant societal burdens regarding medical costs, disability, and lost productivity [1, 2]. The advent of biological disease-modifying antirheumatic drugs (bDMARD) revolutionized RA treatment, enabling clinical remission as an achievable goal for many patients. Consequently, 50%–60% of patients with early RA achieve low disease activity (LDA) or sustained remission [3–6]. This progress has heightened interest in strategies for reducing bDMARD doses and discontinuing bDMARD for patients who maintain LDA. However, the optimal approach to these strategies remains unclear. Establishing evidence-based tapering and discontinuation protocols is an urgent priority.

Although several bDMARD discontinuation trials were conducted [7–10], few studies have investigated extending the interval used in bDMARD treatment [11, 12]. The existing bDMARD discontinuation trials vary widely in terms of bDMARD types examined, patient backgrounds, and inclusion criteria. The question of which patients can safely undergo interval extension or discontinuation remains. Consequently, in clinical practice, decisions regarding the tapering pace and duration are often based on the attending physician’s expertise. Predictors of RA flares during a drug’s dose reduction or discontinuation must also be identified to ensure effective and safe treatment strategies. Addressing these challenges will enhance long-term RA management and contribute to healthcare economics. We conducted this study to identify (*i*) the effects of dose-reduction and discontinuation protocols for bDMARD in patients with stable RA and (*ii*) predictors of flares.

## Patients and Methods

### Study design

This prospective clinical trial enrolled RA patients treated at Kindai University School of Medicine, Department of Hematology and Rheumatology (Osaka, Japan) from 2014 to 2024. Eligible patients met the American College of Rheumatology (ACR) criteria (pre-2009) [13] and the 2010 ACR/EULAR classification criteria [14]. The study targeted RA patients in remission for ≥ 6 months with a stable dose of a bDMARD (infliximab, adalimumab, etanercept, certolizumab, tocilizumab, sarilumab, or abatacept) and a csDMARD, including methotrexate (MTX). Patients were divided into two groups (Suppl. Fig. S1). Allocation was non-randomized and reflected shared decision-making incorporating patient willingness to taper and the treating physician’s judgment. Because joint counts are included by disease activity score (DAS) 28, swollen and tender joints were not used as the eligibility criteria. Propensity score matching was performed using age, sex, disease duration, and baseline DAS28 as primary covariates, with health assessment questionnaire–disability index (HAQ-DI) and C-reactive protein (CRP) additionally included to account for functional status and inflammatory burden, thereby minimizing baseline imbalances between groups. The control group (*n* = 337) comprised RA patients who remained clinically stable and continued standard interval bDMARD therapy without discontinuation during the first 6 months after initiation; they were assessed at 6 months and then followed for up to 24 months on the approved regimen. Baseline systemic glucocorticoids were permitted and were recorded. The Reduction group (*n* = 80) initiated stepwise bDMARD dose-spacing after achieving and maintaining remission (DAS28 < 2.6) for ≥ 6 months; baseline systemic glucocorticoids were not permitted. The Reduction group followed a stepwise dose-reduction protocol, gradually extending dosing intervals before discontinuation. The dose-reduction protocol extended dosing to 1.5-times the standard interval for 6 months, then 2.0-times for another 6 months, followed by discontinuation. This included Phase 1 (12-month interval extension) and Phase 2 (discontinuation). The dosing interval extension protocol, in which the interval was increased from 1.5 to 2.0 times the standard interval, was based on the previous report [15]. The 6-month interval for modifying the dosing schedule was determined with reference to the 2023 EULAR recommendations [6]. DAS28 ≤ 3.2 indicated continuation, whereas DAS28 > 3.2 triggered assessment for possible flare. Rescue therapy-defined as a return to standard-interval bDMARD therapy-could be initiated at the physician’s discretion without waiting 3 months. The primary endpoint defined a flare as DAS28 > 3.2 sustained for more than 3 months. We also conducted analyses that treated the first time DAS28 exceeded 3.2 and the time rescue therapy was instituted as events, and both identified the same event time points.

Our protocol was designed to consider discontinuation following a stepwise taper. To minimize risk, patients were closely monitored, and discontinuation at 12 months was considered only for those who completed tapering without flare and provided consent to withdrawal. To detect the flare early, all patients who entered Phase 2 underwent standardized musculoskeletal ultrasound, assessing gray scale (GS) synovitis and power Doppler (PD) in conjunction with clinical findings and serological biomarkers. In parallel, predictors of rheumatoid arthritis flare—patient background, serological markers (rheumatoid factor [RF], anti-citrullinated peptide antibody [ACPA]), treatment exposures, and joint ultrasound findings—were evaluated, and the flare-free population proportion and flare rates were compared between groups over a 2-year follow-up.

### Clinical evaluations

The study’s primary endpoint was the flare-free population rate in the Reduction group compared to that of the control group. The secondary endpoints were the flare rate, disease activity, functional ability, and joint ultrasound findings. Based on the disease activity categories for the DAS28 [16], we classified the patients as being in remission (DAS28 < 2.6) or with LDA (≥ 2.6 and < 3.2), moderate disease activity (3.2–5.1), or high disease activity (> 5.1). Moderate-to-high disease activity was defined as DAS28 > 3.2. The continuation criteria for the dose-reduction protocol were LDA with DAS28 < 3.2 points.

In this study, drug-free was defined as no use of any bDMARD or csDMARD throughout Phase 2, including MTX. We generated Kaplan–Meier curves with 95% confidence intervals for both the drug-free group (*n* = 11; no csDMARD from the start of Phase 2 to the last observation) and the overall Phase 2 group (*n* = 41). We also stratified by bDMARD class (TNFi, IL-6Ri, CTLA-4Ig) and by MTX use at the time of discontinuation (yes/no). Group differences were compared using the log-rank test. Because strata were small, no covariate adjustment was performed for the stratified comparisons.

The patients’ functional ability was assessed with the Health Assessment Questionnaire Disability Index (HAQ-DI) [17]. DAS28 and HAQ-DI evaluations were performed every 3 months from the study’s initiation. Ultrasound examinations were conducted at the initiation of the dose-interval extension phase and at the start of bDMARD discontinuation. The joint ultrasound examinations were scored on the GS and PD scale by two qualified sonographers blinded to the treatment, with > 10 years’ musculoskeletal ultrasound expertise, and certification in the OMERACT-EULAR synovitis scoring system [18]. All of the joint ultrasound examinations were performed with an ultrasound system (Fujifilm Medical, Tokyo) equipped with a 5- to 18-MHz linear array transducer. The color Doppler settings remained consistent throughout the study, with the Doppler frequency set at 7.5 MHz, the pulse repetition frequency at 0.8 kHz, color priority at 100%, and the Doppler gain adjusted just below the noise level. The overall weighted kappa value was 0.8. Ultrasound synovitis was scored 0–3 per joint for GS and PD (0 = absent, 1 = mild, 2 = moderate, 3 = severe). We assessed 48 joints in total, yielding maximum totals of 0–144 for both GS and PD. Suppl. Table. S1 shows the joint ultrasound evaluation joint sites.

### Safety monitoring

Safety monitoring was performed by the attending physician at each visit. The physicians recorded all adverse events (AEs) and made any necessary treatment adjustments according to the study protocol. Serious AEs were defined as AEs if they resulted in infection requiring hospitalization, infusion or infection reaction, a cardiovascular event, malignancy, or death.

### Statistical analyses

This study was designed as an exploratory analysis. The sample size was determined by the number of patients who met the eligibility criteria and completed the 12-month follow-up period. Of these, 71 patients who agreed to bDMARD dose reduction were propensity score-matched 1:1 with 71 patients selected from 337 individuals in the control group who continued standard RA therapy. Matching was based on age, sex, disease duration, baseline DAS28, and additional covariates such as baseline HAQ-DI and CRP levels, to ensure comparability between groups. Patients with critical missing baseline data were excluded. Continuous variables are presented as medians with interquartile ranges (IQR) or means with standard deviations (SD), and categorical variables as proportions. Standardized mean differences (SMDs) were calculated as standardized mean differences. Thresholds for interpreting |SMD|: <0.10, good balance; 0.10–0.20, small; 0.20–0.50, moderate; >0.50, large. Between-group comparisons were performed using the Mann–Whitney U-test for continuous variables and either Fisher’s exact test or the chi-squared test for categorical variables. To evaluate the association between treatment group and flare occurrence, both unadjusted and adjusted odds ratios (OR) with 95% confidence intervals (CIs) were calculated. Multivariate logistic regression models were adjusted for age, sex, and disease duration as potential confounders. Subgroup analyses and interaction testing were conducted to explore effect modification, with subgroups defined by the positivity of RF and ACPA, and MTX use. Interaction terms were included in the logistic regression models and assessed using Wald tests. Kaplan–Meier survival analyses were used to assess time to the flare (flare-free survival) and bDMARD flare-free population. Patients were censored at their last available follow-up, and group differences were tested using log-rank tests. Baseline balance was assessed using absolute standardized mean differences (SMDs). Variables with SMD ≥ 0.15 (CRP and power Doppler score) were prespecified for covariate adjustment in outcome models.All statistical analyses were performed using JMP Pro 18 (SAS Institute Inc., Cary, NC, USA) for regression modeling and summary statistics, and GraphPad Prism (GraphPad Software Inc., San Diego, CA, USA) for survival analyses and graphical presentation. A two-sided p-value of < 0.05 was considered statistically significant.

## Results

### Patient characteristics

Table 1 summarizes the baseline clinical and laboratory characteristics of the 417 RA patients divided into bDMARD Reduction group and control groups, both before and after propensity score matching. Before the propensity score matching, the control group demonstrated a significantly longer mean disease duration compared to the Reduction group (44.0 months [13.0–141.0] vs. 19.0 months [9.0–60.0], *p* < 0.01). DAS28 including tender and swollen counts (TJC and SJC), patient and physician visual analogue scale (PtVAS and DrVAS) was significantly higher in the control group versus the Reduction group (2.9 ± 1.2 mm vs. 1.9 ± 0.7 mm, *p* < 0.05). However, after propensity matching, these disease duration and disease activity such as DAS28 including TJC, SJC, PtVAS, and DrVAS differences were balanced, resulting in comparable distributions across the groups. Similarly, CRP and Matrix metalloproteinase-3 (MMP-3) was notably higher in the control vs. bDMARD Reduction group before matching (0.4 mg/dL [0.3–0.5] vs. 0.01 mg/dL [−0.2-0.3] and 134.7 ng/mL [116.6–152.8] vs. 86.1 ng/mL [46.8–125.4.8.4], *p* < 0.05), but this discrepancy was minimized after matching. Remission duration at baseline was comparable between groups both before matching (median 15.0 [6.1–19.5] vs. 26.5 [17.2–31.5] months) and after matching (median 15.8 [6.2–18.3] vs. 19.9 [10.1–31.1] months). In addition, no patients with a history of flares were identified at enrollment (data not shown). Our analyses also revealed that the RF and ACPA positivity was significantly higher in the control vs. bDMARD Reduction group before matching (79.7% vs. 50.8% and 83.9% vs. 57.1%, *p* < 0.05), while RF and ACPA titers were similar in the groups before and after matching.

**Table 1:** The baseline clinical and laboratory data of the patients with rheumatoid arthritis

PS matching status	Before PS matching			After PS matching			
	bDMARD Reduction *n* = 80	Control *n* = 337	SMD		bDMARD Reduction *n* = 71	Control *n* = 71	SMD
Age, yrs	58.7 ± 17.3	61.7 ± 15.4	0.18		58.9 ± 17.4	58.9 ± 17.3	0.00
Female (%)	77.8	77.9	0.0		79.0	77.4	0.04
Disease duration, mos.	19.0 [9.0–60.0]	44.0 [13.0–141.0]**	0.35		21.0 [8.8–61.0]	22.0 [9.8–55.8]	0.03
Time from bDMARDs introduction to start of drug reduction, mos.	24.0 [12.0–36.0]	–	–		22.0 [11.0–36.5]	–	–
Remission duration, mos	15.0 [6.1–19.5]	26.5 [17.2–31.5]	0.28		15.8 [6.2–18.3]	19.9 [10.1–31.1]	0.14
RF, (%)/IU/mL	50.8, 77.7 [18.7–136.8]	79.7*, 135.4 [108.3–162.6]	0.64/0.85		48.4, 21.3 [0.2–84.3]	40.3, 21.3 [6.0–64.3]	0.14/0.00
ACPA, (%)/U/mL	57.1, 20.1 [0.2–81.1]	83.9*, 64.5 [13.3–284]	0.61/0.30		58.1, 20.1 [0.2–81.1]	66.1, 22.0 [0.0–95.4]	0.12/0.03
CRP, mg/dL [IQR]	0.01 [–0.2–0.3]	0.4 [0.3–0.5]*	1.38		0.02 [0.01–0.06]	0.02 [0.01–0.06]	0.00
ESR, mm/hr [IQR]	9.6 [2.1–5.5]	17.9 [0.98–17.69]	0.44		8.0 [5.8–11.3]	6.0 [3.0–13.3]	0.17
MMP-3, ng/mL [IQR]	86.1 [46.8–125.4]	134.7 [116.6–152.8]*	0.57		50.0 [38.1–68.3]	58.6 [40.2–123.6]	0.19
Tender joints, range 0–28 [IQR]	0.6 ± 1.1	2.1 ± 0.2*	1.55		0.6 ± 1.1	0.8 ± 1.8	0.12
Swollen joints, range 0–28 [IQR]	0.3 ± 0.3	1.7 ± 0.2*	5.28		0.3 ± 0.7	1.0 ± 2.8	0.30
Patient visual analogue scale, 0–100 mm	9.1 [3.8–14.3]	26.0 [23.7–28.3]*	1.14		5.0 [0.0–12.3]	8.0 [2.0–13.0]	0.35
Physician visual analogue scale, 0–100 mm	5.6 [3.6–7.7]	22.8 [20.5–25.1]*	5.33		2.0 [0.0–10.0]	7.5 [2.0–10.3]	0.81
DAS28	1.9 ± 0.7	2.9 ± 1.2*	1.02		1.8 ± 0.1	1.8 ± 0.1	0.00
bDMARDs: 1st/2nd/3rd/others (%)	84.1/14.3/1.6/0.0	67.2/22.4/5.3/5.1*	0.4/0.21/0.2/0.33		83.4/14.5/1.6/0.0	82.3/17.7/0.0/0.0	0.03/0.09/0.18/0.0
TNFi/IL-6Ri/CTLA4-Ig (%)	49.2/41.3/9.5	45.4/34.3/20.3	0.08/0.14/0.31		48.4/41.9/9.7	43.6/50.0/6.4	0.10/0.16/0.12
Infliximab/certolizumab pegol/ etanercept/adalimumab (%)	11.1/19.0/7.9/11.1	9.9/6.9/11.0/8.7	0.04/0.37/0.06/0.08		11.3/19.4/8.1/9.7	9.7/11.3/9.7/9.7	0.05/0.23/0.06/0.0
Tocilizumab/sarilumab (%)	23.8/17.5	19.4/14.9	0.11/0.07		24.1/17.7	19.4/30.7	0.11/0.31
Abatacept (%)	9.6	20.3	0.3		9.7	6.5	0.12
No. of csDMARD	1.1 ± 0.1	1.2 ± 0.0	0.53		0.9 ± 0.1	1.1 ± 0.1	0.34
MTX (%), mg/week [IQR]	52.4, 4.0 [0.0–8.0]	58.2, 4.0 [0.0–8.0]	0.12/0.0		51.6/3.0 [0.0–8.0]	67.7*/0.0 [0.0–8.0]	0.33/0.51
SASP/IGU/BUC/TAC/none (%)	4.8/27.0/0.0/6.4/25.4	28.1/22.4/6.3/9.0/15.5*	0.66/0.11/0.37/0.1/0.25		4.8/27.4/0.0/6.5/25.8	23.0*/24.2/4.9/3.3/19.4	0.55/0.07/0.32/0.15/0.15
Glucocorticoid (%), mg/day	–	40.3/0.0 [0.0–3.0]	–		-	32.3/0.0 [0.0–2.0]	–
Steinbrocker stage I/II/III/IV (%)	73.0/19.0/4.8/3.2	53.7/18.8/11.7/15.7*	0.41/0.01/0.25/0.44		72.6/19.4/4.8/3.2	67.2/23.0/6.6/3.3	0.12/0.09/0.08/0.01
Steinbrocker class 1/2/3/4 (%)	88.9/4.8/6.3/0.0	31.8/41.8/24.9/1.5*	1.44/0.97/0.53/0.17		88.7/4.8/6.5/0.0	41.9/41.9/16.2/0.0	1.13/0.98/0.31/0.0/0.28
HAQ-DI, range 0–3	0.0 [0.0–0.3]	0.4 [0.0–1.0]**	0.73		0.0 [0.0–0.3]	0.1 [0.0–0.6]	0.28

Values are median [25th–75th centiles] or mean (SD), unless otherwise indicated. *p < 0.05, **p < 0.01. *PS* propensity score, *SMD* standardized mean difference, *ACPA* anti-citrullinated peptide antibody, *bDMARD* biological disease-modifying antirheumatic drug, *BUC* bucillamine, *CRP* C-reactive protein, *csDMARD* conventional synthetic disease-modifying antirheumatic drug, *CTLA-Ig* cytotoxic T-lymphocyte-associated protein 4-immunoglobulin, *ESR* erythrocyte sedimentation rate, *HAQ-DI* Health Assessment Questionnaire-Disability Index, *IGU* iguratimod, *IL-6Ri* interleukin-6 receptor inhibitor, *IQR* interquartile range, *mos*,: months, *MTX* methotrexate, *RF* rheumatoid factor, *SASP* sulfasalazine, *TAC* tacrolimus, *TNFi* tumor necrosis factor inhibitor

The treatment patterns differed: sulfasalazine and bucillamine were more commonly combined with control vs. bDMARD Reduction group (23.0% vs. 4.8% and 4.9% vs. 0.0%, *p* < 0.05). bDMARD Reduction group was more commonly used as a first-line bDMARD (84.1% vs. 67.2%, *p* < 0.05). Before matching, there was no difference in MTX use between the groups; however, after matching, the control group showed an increased rate of MTX use compared to the bDMARD Reduction group (67.7% vs. 51.6%, *p* < 0.05). Before matching, the bDMARD reduction group showed significantly lower Steinbrocker stage and class distributions as well as significantly lower HAQ-DI values compared to the control group (stage: 73.0/19.0/4.8/3.2 vs. 53.7/18.8/11.7/15.7; class: 88.9/4.8/6.3/0.0 vs. 31.8/41.8/24.9/1.5; HAQ-DI: 0.0 [0.0–0.3] vs. 0.4 [0.0–1.0], *p* < 0.01). After matching, no significant differences were observed between the two groups in terms of Steinbrocker stage, class, or HAQ-DI. After matching, these treatment distributions balanced, and we conducted the further statistical analyses on adjusted groups. Baseline balance by censoring status. In Suppl. Table S3, absolute SMDs were generally small (most < 0.10), indicating good baseline comparability between withdrawal-censored and observed patients. Two covariates in CRP and the Power Doppler score showed modest imbalances (SMDs ≥ 0.15) both were included as covariates in multivariable models, and the main results were unchanged.

### Flare-free population and flare rates

**Fig. 1:**
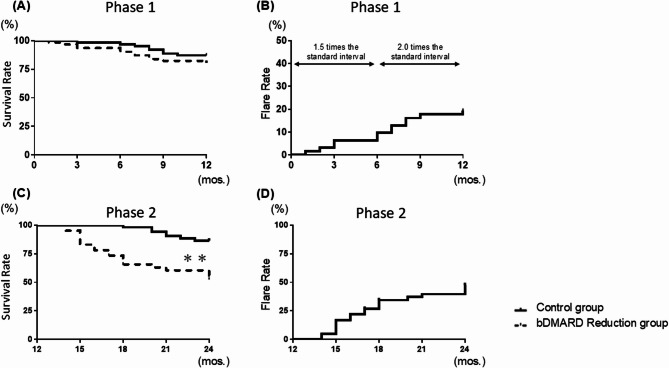
The flare-free population rate and flare rate during the dose-interval extension and discontinuation phases. **A** The overall flare-free population rate, including all reasons for treatment discontinuation in the control group receiving standard therapy versus the bDMARD Reduction group, during Phases (1) (**B**) Flare rates in the bDMARD Reduction group during Phase 1, categorized by dose intervals of 1.5 and 2.0 times the standard intervals. **C** The overall flare-free population rate, including all reasons for treatment discontinuation in the control group receiving standard therapy versus the bDMARD Reduction group, during Phases (2) (**D**) Flare rates following bDMARD discontinuation during Phase 2. bDMARD: biological disease-modifying anti-rheumatic drug, DAS28: Disease Activity Score of 28 joints, HAQ-DI: Health Assessment Questionnaire-Disability Index

We demonstrated the groups’ flare-free population and flare rates during Phase 1 and 2 in Fig. 1. In Phase 1, no significant differences in the overall flare-free population rate (including all reasons such as AEs and patient withdrawal for treatment discontinuation) were observed between the control group and Reduction group at 6 months (96.8% vs. 90.3%) and 12 months (87.1% vs. 80.6%) (Fig. 1A). In Phase 2, when the bDMARD was eventually discontinued, the flare-free population rate at 24 months was significantly lower in the Reduction patients versus the controls (52.5% vs. 86.6%), with a hazard ratio (HR) for flares at 4.6 (95%CIs: 2.2–11.0, *p* < 0.001) (Fig. 1C). Figure 1 (B and D) illustrates the flare rates of RA disease activity during Phases 1 and 2, excluding AEs and patient withdrawals, for the Reduction and control groups.

To further investigate the association between dose intervals and flare rates in the Reduction group, we analyzed the flare rates by dividing Phase 1 into intervals of 1.5 times and 2.0 times the standard dose interval (Fig. 1B). The results revealed a gradual increase in flare rates as the dose interval extended from 1.5 to 2.0-times the standard, indicating a dose-dependent increase in flare frequency during the interval-extension phase (6 months: 9.7%, 12 months: 19.4%). In Phase 1, flare rates by the degree of interval extension were as follows. For tumor necrosis factor inhibitors (TNFi): no flare 73.5% (25/34), flare at a 1.5-time interval 11.8% (4/34), and flare at a 2.0-time interval 14.7% (5/34). For interleukin-6 receptor inhibitors (IL-6Ri): no flare 80.0% (24/30), flare at a 1.5-time interval 10.0% (3/30), and flare at a 2.0-time interval 10.0% (3/30) (Suppl. Fig. S3A). Figure 1D depicts the flare rates during Phase 2 in the Reduction group after treatment discontinuation. Within 12 months following bDMARD discontinuation, nearly half of the patients experienced flares (47.5%). Kaplan–Meier curves indicated lower flare-free survival in the drug-free group compared with the overall Phase 2 group (Suppl. Fig. S3B). At 24 months, the estimated flare-free proportion was 18.2% in the drug-free group versus 52.4% in the overall group (log-rank *p* = 0.07, HR = 2.50, 95% CI: 0.93–6.80).

### Disease activity and function

**Fig. 2:**
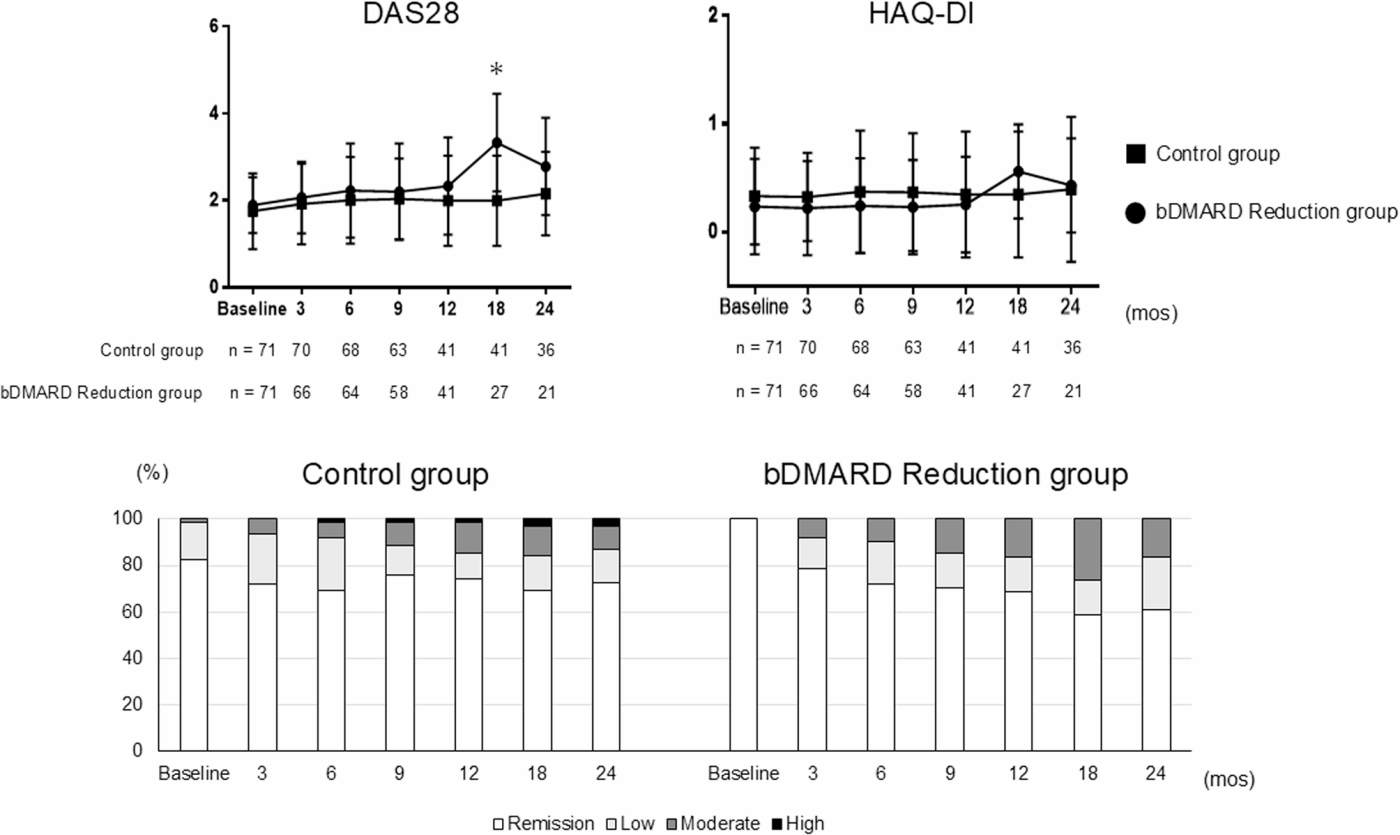
The disease activity and functional outcomes during the dose-interval extension and discontinuation phases. Changes in the DAS28 scores and HAQ-DI scores for the control and bDMARD Reduction groups during the study period. Comparison of the groups’ DAS28 scores during the study period. The disease activity and DAS28 criteria were categorized as follows. The DAS28 criteria: high: >5.1, moderate: 3.2–5.1 (moderate-to-high: >3.2), low: ≥2.6 to ≤ 3.2, remission: <2.6

The results comparing disease activity and functional disability between the two groups at baseline and at 3, 6, 9, 12, 18, and 24 months, based on DAS28 and HAQ-DI, are presented in Fig. 2. From baseline to 3, 6, 9, 12, and 24 months, no significant changes between-group differences in DAS28 were observed. However, the DAS28 scores showed a significant increase in the Reduction group versus the controls at 18 months (2.9 ± 1.2 vs. 3.3 ± 1.1, *p* < 0.05). HAQ-DI scores consistently showed no significant differences in the Reduction group versus the controls from baseline to 24 months. The 18-month spike is attributable to clinical deterioration in a subset of the bDMARD Reduction group, followed by rescue intervention or discontinuation. This selective attrition altered the composition of the risk set toward clinically stable patients (DAS28 ≤ 3.2), resulting in a subsequent decline in the conditional (observed) mean DAS28 and HAQ-DI.

Furthermore, the 24-month evaluation of LDA (including remission) and moderate-to-high disease activity in the two groups. In the control group, the proportion of patients achieving LDA increased from 98.4% at baseline to 88.7% at 9 months and stabilized at 87.1% by 24 months. In the Reduction group, 91.8% of patients achieved LDA at 3 months, but this decreased to 83.6% at 24 months.

### Background of the non-flare and flare patients in the reduction group

Table 2 presents a comparative analysis of non-flare and flare patients in the Reduction group across both phases. Baseline characteristics, disease activity, and treatment details of 71 RA patients were analyzed for flare incidence during Phase 1.

**Table 2:** Background, laboratory data, and treatment details at baseline in phase 1 and phase 2 for the bDMARD reduction group

	Phase 1 (baseline)		Phase 2 (12 mos.)	
	Non-flare *n* = 55	Flare *n* = 16	Non-flare *n* = 21	Flare *n* = 20
Age, yrs	59.5 ± 17.7	56.5 ± 16.3	60.7 ± 15.7	53.7 ± 20.6
Female (%)	83.7	61.5*	71.4	90.0
Disease duration, mos.	17.0 [8.0–67.0]	37.0 [16.5–57.5]	12.0 [5.5–29.5]	27.5 [11.3–71.5] ^#^
Time from bDMARDs introduction to start of drug reduction, mos.	23.0 [12.0–36.0]	26.0 [12.5–36.0]	22.5 [11.8–30.3]	27.0 [14.0–42.5]
Remission duration, mos	15.7 [7.2–18.3]	15.5 [7.1–20.2]	15.8 [6.8–17.1]	15.5 [7.2–19.5]
RF (%)/titer	50.0, 15.0 [8.0–46.5]	61.5, 27.0 [7.0–269.5]	42.9, 10.0 [6.5–25.0]	65.0, 29.0 [10.8–137.5] ^#^
ACPA (%)/titer	55.1, 17.8 [0.0–68.5]	69.2*, 56.2 [0.3–438.0]*	47.6, 5.0 [0.0–63.1]	70.0^#^, 54.7 [8.0–169.7] ^#^
CRP, mg/dL [IQR]	0.02 [0.01–0.05]	0.05 [0.01–0.13]	0.0 [0.0–0.1]	0.1 [0.0–0.2]
ESR, mm/hr [IQR]	8.0 [5.0–10.5]	8.0 [7.5–12.5]	8.5 [6.0–19.5]	12.0 [8.0–17.0]
MMP-3, ng/mL [IQR]	47.5 [37.4–64.3]	66.3 [47.6–98.0]	51.2 [43.3–91.3]	49.6 [41.8–63.3]
Tender joints, range 0–28 [IQR]	0.6 ± 1.1	0.8 ± 1.2	0.2 ± 0.4	0.7 ± 1.4
Swollen joints, range 0–28 [IQR]	0.2 ± 0.5	0.8 ± 1.2*	0.1 ± 0.2	0.2 ± 1.4
Patient visual analogue scale, 0–100 mm	4.0 [0.0–10.0]	13.0 [0.5–17.5]	1.0 [0.0–5.0]	2.0 [0.0–5.0]
Physician visual analogue scale, 0–100 mm	2.0 [0.0–6.0]	9.0 [0.0–17.5]	0.0 [0.0–5.0]	0.0 [0.0–2.5]
DAS28	1.8 ± 0.6	2.2 ± 0.6*	1.9 ± 0.1	2.0 ± 0.1
bDMARDs: 1st/2nd/3rd/others (%)	87.8/10.2/2.0/0.0	69.2/30.8/0.0/0.0	100.0/0.0/0.0/0.0	85.7/14.3/0.0/0.0
TNFi/IL-6Ri/CTLA4-Ig (%)	45.2/42.9/11.9	57.1/38.1/4.8	47.4/47.4/5.3	50.0/40.0/10.0
Infliximab/certolizumab pegol/ etanercept/adalimumab (%)	7.1/16.7/9.5/11.9	19.1/23.8/4.8/9.5	15.8/15.8/5.3/10.5	5.0/20.0/10.0/15.0
Tocilizumab/sarilumab (%)	22.7/16.7	23.4/19.1	21.1/26.3	25.0/15.0
Abatacept (%)	11.9	4.8	5.3	10.0
No. of csDMARD	1.0 ± 0.6	0.7 ± 0.6	1.1 ± 0.2	0.6 ± 0.1*
MTX (%), mg/week [IQR]	51.0, 2.0 [0.0–8.0]	53.9, 4.0 [0.0–9.0]	71.2, 6.0 [0.0–9.0]	33.3*, 0.0 [0.0–4.0]*
SASP/IGU/BUC/TAC/none (%)	4.1/32.7/0.0/8.2/22.5	7.7/7.7/0.0/0.0/38.5*	25.0/23.1/5.8/7.7/11.5	9.5/22.7/0.0/4.8/40.9*
Steinbrocker stage I/II/III/IV (%)	75.5/18.4/4.1/2.0	61.5/23.1/7.7/7.7	77.6/14.3/6.1/2.0	66.7/16.7/8.3/8.3
Steinbrocker class 1/2/3/4 (%)	89.8/4.1/6.1/0.0	84.6/7.7/7.7/0.0	62.2/28.9/8.9/0.0	85.7/14.3/0.0/0.0
HAQ-DI, range 0–3	0.0 [0.0–0.4]	0.1 [0.0–0.2]	0.0 [0.0–0.1]	0.0 [0.0–0.5]

Values are median [25th–75th centiles] or mean (SD) unless otherwise indicated. Non-flare vs. flare in Phase 1; *p < 0.05, **p < 0.01 and Non-flare vs. flare in Phase 2; ^#^*p* < 0.05. *ACPA* anti-citrullinated peptide antibody, *bDMARD* biological disease-modifying antirheumatic drug, *BUC* bucillamine, *CRP* C-reactive protein, *csDMARD* conventional synthetic disease-modifying antirheumatic drug, *CTLA-Ig* cytotoxic T-lymphocyte-associated protein 4-immunoglobulin, *DAS28* Disease Activity Score in 28 joints, *ESR* erythrocyte sedimentation rate, *HAQ-DI* Health Assessment Questionnaire-Disability Index, *IGU* iguratimod, *IL-6Ri* interleukin-6 receptor inhibitor, *IQR* interquartile range, *mos*.: months, *MTX* methotrexate, *RF* rheumatoid factor, *SASP* sulfasalazine, *TAC* tacrolimus, *TNFi* tumor necrosis factor inhibitor, *tsDMARD* targeted synthetic disease-modifying antirheumatic drug

At baseline, DAS28 values were low in both groups, indicating remission, but were significantly lower in non-flare patients (mean 1.8 ± 0.6 vs. 2.2 ± 0.6, *p* < 0.05). No significant age differences were found, but female proportion was higher in non-flare patients (83.7% vs. 61.5%, *p* < 0.05). Disease duration was shorter in non-flare patients (median 17.0 months [8.0–67.0] vs. 37.0 months [16.5–57.5]). ACPA positivity and titers were significantly lower in non-flare patients (55.1% vs. 69.2%; 17.8 U/mL [0.0–68.5] vs. 56.2 U/mL [0.3–438.0], *p* < 0.05), and RF titers also showed a downward trend (42.9% vs. 65.0%; 10.0 IU/mL [6.5–25.0] vs. 29.0 IU/mL [10.8–137.5]).

In Phase 2, 41 patients who discontinued treatment were analyzed. DAS28 did not differ between flare and non-flare patients. However, disease duration was significantly shorter in non-flare patients (median 12.0 months [5.5–29.5] vs. 27.5 months [11.3–71.5], *p* < 0.01), though bDMARD treatment duration before dose reduction was similar.

ACPA positivity and titers remained lower in non-flare patients (47.6% vs. 70.0%; 5.0 U/mL [0.0–63.1] vs. 54.7 U/mL [8.0–169.7], *p* < 0.01). RF positivity and titers were also lower (42.9% vs. 65.0%; 10.0 IU/mL [6.5–25.0] vs. 29.0 IU/mL [10.8–137.5], *p* < 0.01). Non-flare patients received more csDMARD types (1.1 ± 0.2 vs. 0.6 ± 0.1, *p* < 0.05) and had higher MTX use and doses (71.2% vs. 33.3% and 6.0 mg/week [0.0–9.0] vs. 0.0 mg/week [0.0–4.0], *p* < 0.05). Flare patients were significantly more likely to be without csDMARD treatment (40.9% vs. 11.5%, *p* < 0.05).

### Flare rates after discontinuation by mode of action and individual bDMARD

In Phase 2, flare rates following bDMARD discontinuation were analyzed by MTX use within each biologic class (Suppl. Table. S2). After discontinuation, overall flare rates were 33.3% for TNFi (7/21), 52.9% for IL-6Ri (9/17), and 33.3% for CTLA-4 Ig (1/3). At the individual-agent level, flare rates were 50.0% for etanercept and sarilumab, and 44.4% for tocilizumab. The mean time to relapse after discontinuation varied among agents, with longer intervals observed for adalimumab (14.0 ± 15.6 months) and certolizumab (12.5 ± 16.4 months), whereas abatacept showed a relatively short time to relapse (2.5 ± 0.7 months). In Phase 2, flares were more common without methotrexate (MTX), as the flare rate was 25.0% (3/12) with MTX and 44.4% (4/9) without MTX for TNFi, whereas it was 17.0% (3/8) with MTX and 66.7% (6/9) without MTX for IL-6Ri, and it was 50.0% (1/2) with MTX and 0% (0/1) without MTX for CTLA-4Ig.

### Flare-free population rates by serological factors, mtx use, and mode of action in the bDMARD interval extension and discontinuation protocol

**Fig. 3:**
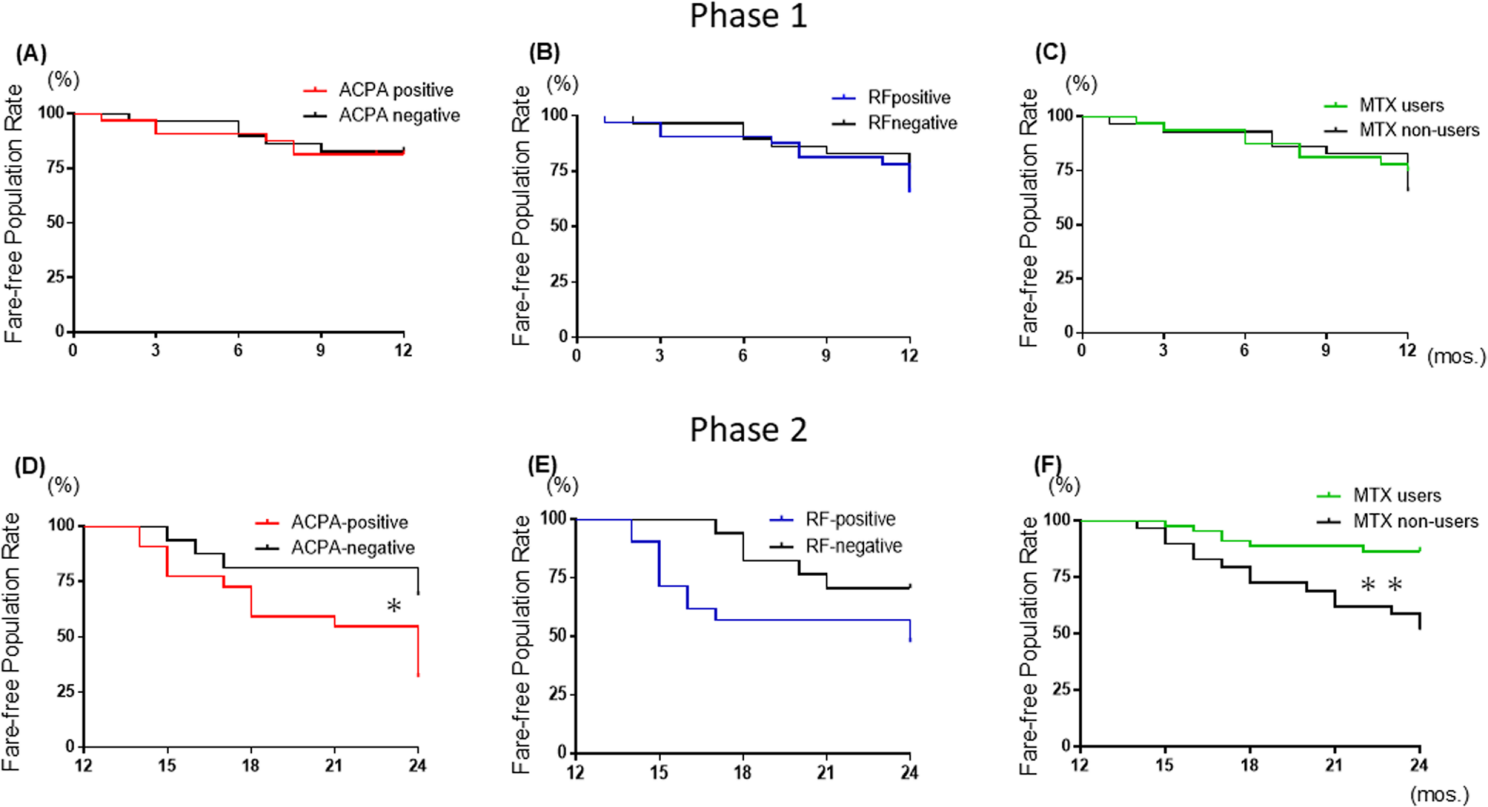
Flare-free population rates by bDMARD subgroups during the dose-interval extension and discontinuation phases. Panels **(A–C)** show the flare-free population rates in Phase 1, and panels **(D–F)** illustrate the flare-free population rates in Phase 2. Flare-free population rates by ACPA **(A **and** D)** and RF positivity **(B **and** E)** in Phase 1 and 2. Flare-free population rates by the concomitant use/non-use of MTX. **(C **and** F)** in Phase 1 and 2 as defining the baseline to 12 months as Phase 1 (the interval extension phase) and the period after 12 months as Phase 2 (the discontinuation phase). The significance of differences was assessed using the log-rank test. ACPA; anti-citrullinated protein antibodies, MTX: methotrexate

We evaluated the flare-free population rates in the bDMARD Reduction group based on three factors: ACPA positivity, RF positivity, and MTX use. Figure 3 presents the results. Panels A–C show the flare-free population rates in Phase 1, and panels D–F illustrate the flare-free population rates in Phase 2, based on each factor in relation to flares. In Phase 1, no significant differences in flare-free population rates were observed based on ACPA status, RF status, or MTX use (Fig. 2A–C).

In contrast, during Phase 2, ACPA-positive patients exhibited significantly lower flare-free population rates compared with their ACPA-negative counterparts (31.2% vs. 68.8%) with a HR for flares at 2.7 (95%CIs: 1.2–6.7, *p* < 0.05) (Fig. 2D). Similarly, in Phase 2, RF-positive patients showed a trend toward lower flare-free population rates compared with RF-negative patients; however, the difference was not statistically significant (Fig. 2E).

When examining the impact of MTX treatment on flare-free population, a significant difference in flare-free population rates was observed between MTX users and non-users during Phase 2, with non-users showing lower flare-free population (38.0% vs. 17.0%) with a HR for flares at 4.2 (95%CIs: 1.8–11.5, *p* < 0.001) (Fig. 2F).

### Gray scale and power doppler ultrasound findings

**Fig. 4:**
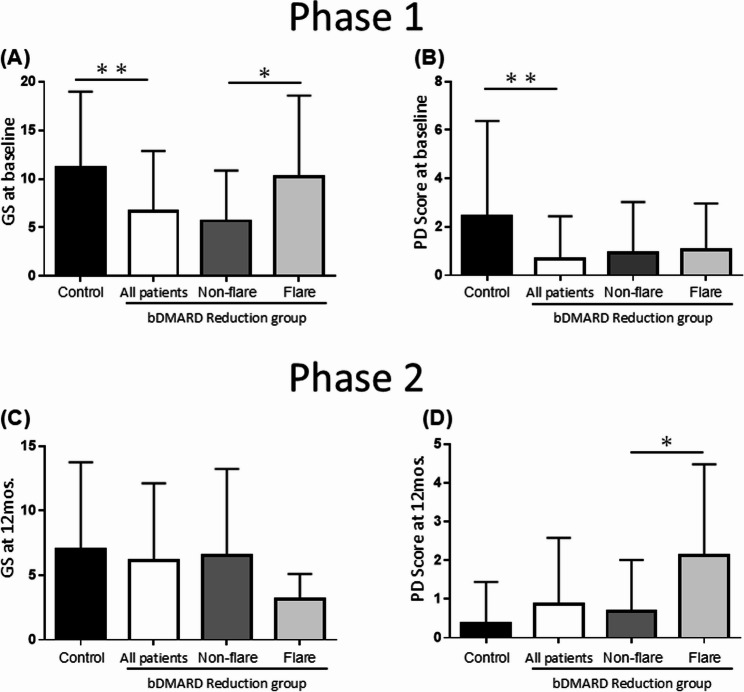
The assessment of flare risk based on joint ultrasound findings in the dose-interval extension and discontinuation phases. The gray scale (GS) and power Doppler (PD) scale ultrasound scores at baseline for the control and bDMARD Reduction groups during Phases 1 and 2 are shown

Figure 4 illustrates the baseline GS and PD ultrasound scores divided into Phase 1 (at baseline) and 2 (at 12mos.) analyses. In Phase 1, both the GS and PD scores were significantly higher in the control group versus the other groups (*p* < 0.001). Notably, the GS were significantly different between the flare and non-flare patients (GS: 10.2 ± 8.3 vs. 5.7 ± 5.2, *p* < 0.05). In Phase 2, the GS and PD scores were no differences in the control group compared to the Reduction group, and the PD scores were significantly different between the patients with flares and those without flares (2.1 ± 2.4 vs. 0.7 ± 1.3, *p* < 0.05).

### The treatment course after flares in the bDMARD dose-reducing protocol

The course of the patients’ DAS28 scores after they resumed treatment with bDMARD at a standard dose interval after a flare is illustrated in Suppl. Fig.S2. The mean DAS28 score of these patients was 4.1 ± 1.0 when standard therapy was resumed, 2.7 ± 0.9 at 3 months, 2.8 ± 1.0 at 6 months, 2.6 ± 1.2 at 12 months, and 1.8 ± 0.4 at 24 months (Suppl. Fig. S2A). After resuming standard therapy, 100% of these patients exhibited moderate-to-high disease activity. This proportion decreased to 14.8% after 3 months and further plateaued at 6 and 12 months but disappeared at 24 months (Suppl. Fig. S2B). Remission (DAS28 < 2.6) was achieved in 48.2% of patients at 3 and 6 months, 51.8% at 12 months, and 92.3% at 24 months (numbers at risk provided). Thus, the remission proportion exceeded 50% by 12 months, indicating that remission was generally regained within months after resuming standard-interval therapy.

### The results of the multivariate logistic regression for the prediction of flare

Next, a multivariate logistic regression model was conducted to identify predictors of disease flare in RA patients on the bDMARD dose reduction protocol (Table 3). We analyzed predictors of flare for Phases 1 and 2 in a model including the autoantibody status at baseline (RF and ACPA), the control group as the standard treatment, and the Reduction group plus other disease-specific variables (MTX use, GS and PD scores on joint ultrasonography). In Phase 1, only the GS findings on joint ultrasound (Wald χ²=4.22, *p* = 0.04, OR 1.06) were identified as predictors for disease flares. No significant differences were observed between the control group and interval extension in the Reduction group. In Phase 2, the following factors were identified as significant predictors of disease flare: ACPA positivity (Wald χ²=4.73, *p* = 0.03, OR 3.59), bDMARD discontinuation (Wald χ²=4.1, *p* = 0.04, OR 0.2), MTX non-use (Wald χ²=11.9, *p* = 0.01, OR 1.69), and PD scores on joint ultrasound (Wald χ²=1.56, *p* = 0.04, OR 1.34).

**Table 3:** Results of the multivariate logistic regression for the prediction of flare

	Phase 1								Phase 2							
	Unadjusted				Adjusted				Unadjusted				Adjusted			
	Wald	*p*-value	OR	95%CIs	Wald	*p*-value	OR	95%CIs	Wald	*p*-value	OR	95%CIs	Wald	*p*-value	OR	95%CIs
Positive RF	0.01	0.93	1.02	0.62–1.68	0.01	0.96	1.01	0.61–1.67	1.07	0.3	1.65	0.64–4.27	0.28	0.6	1.43	0.19–2.64
Positive ACPA	0.19	0.66	1.12	0.68–1.84	0.12	0.73	1.1	0.64–1.87	1.7	0.19	1.89	0.73–4.93	4.73	0.03	3.59	1.13–11.35
Control vs. bDMARD Reduction Group	0.01	0.94	1.01	0.75–1.36	0.01	0.94	1.01	0.75–1.37	4.76	0.03	0.27	0.08–0.87	4.1	0.04	0.2	0.04–0.95
MTX non-intake	0.01	0.98	0.99	0.61–1.63	0.01	0.98	0.99	0.563–1.75	11.9	0.01	1.58	2.87–4.55	11.9	0.01	3.69	2.89–4.26
Gray scale	4.12	0.02	2.35	1.19–4.95	4.22	0.04	1.06	1.01–1.28	0.32	0.57	1.03	0.91–1.15	0.07	0.79	0.71	0.84–1.12
Power Doppler scale	0.02	0.89	1.02	0.71–1.38	2.37	0.12	1.12	0.95–1.27	1.86	0.03	1.52	1.12–3.12	1.56	0.04	1.34	1.00–2.58

Adjusted for baseline age, disease duration, and sex. ACPA, and bDMARD

*ACPA* anti-citrullinated peptide antibody, *bDMARD* biological disease-modifying antirheumatic drug, *CIs* confidence intervals, *MTX* methotrexate, *OR* odds ratio, *RF* rheumatoid factor

### Safety summary

The safety of the bDMARD dose reduction strategies was compared with the standard treatment protocols by an analysis of AEs leading to treatment discontinuation (Table 4.). Data was collected over the two phases: Phase 1 included 337 patients in the control group and 71 in the Reduction group, and Phase 2 included 268 control group patients and 41 in the Reduction group. In Phase 1, the incidence of AEs leading to treatment discontinuation was low. Infections caused discontinuation in 1.8% of the control group and 0.0% of the Reduction group. The malignancy and mortality rates were also lower in the Reduction group (0.3% and 0.6% in the control group vs. 0.0% in the Reduction group). In Phase 2, the infection rates were 1.1% in the control group and 0% in the Reduction group. No malignancies or deaths occurred in the Reduction group, whereas malignancies affected 0.4% of the control group. No cases of infusion/injection reactions or cardiovascular events were reported in either group during either phase.

**Table 4:** Adverse events leading to treatment discontinuation during the study observation

	**Phase 1**			**Phase 2**		
	**Control**	**bDMARD** **Reduction**	**Difference** **(%)**	**Control**	**bDMARD** **Reduction **	**Difference** **(%)**
n (%)	71	71		54	41	
Infection	6 (8.4)	0 (0.0)	1.8	3 (5.6)	0 (0.0)	1.1
Infusion and injection reaction	0 (0.0)	0 (0.0)	0.0	0 (0.0)	0 (0.0)	0.0
Cardiovascular event	0 (0.0)	0 (0.0)	0.0	0 (0.0)	0 (0.0)	0.0
Malignancy	1 (1.4)	0 (0.0)	0.3	1 (1.9)	0 (0.0)	0.4
Death	2 (2.8)	0 (0.0)	0.6	0 (0.0)	0 (0.0)	0.0

*bDMARD* biological disease-modifying antirheumatic drug

## Discussion

This study demonstrated that in RA patients with sustained remission, extending bDMARD dosing intervals led to a slight but not significant increase in flare rates compared to standard therapy. However, even among those who successfully extended the interval, flares occurred after bDMARD discontinuation, necessitating careful evaluation. Notably, this strategy showed safety benefits, reducing AEs during both the interval extension and discontinuation phases.

In Phase 1, most patients maintained stable disease activity during interval extension. In Phase 2, full discontinuation significantly increased flare rates, with ~ 50% of patients experiencing a flare within the first year. These results align with existing reports suggesting dose reduction is safer than discontinuation in minimizing flare risks [5, 10, 19]. However, treatment outcomes post-flare remains crucial. An earlier study reported that > 80% of patients regained disease control after resuming treatment [20]. In contrast, csDMARD discontinuation showed lower success rates, with only ~ 50% regaining control [21, 22]. Moreover, stopping bDMARD without MTX was linked to very high flare rates [23]. During bDMARD de-escalation (dose reduction or discontinuation), MTX may reduce the risk of flare. Across all bDMARD classes—particularly with IL-6Ri—the absence of MTX was associated with more flares. These findings support the EULAR recommendation to taper only in patients who are glucocorticoid-free and in sustained remission for at least 6 months, and to taper while maintaining concomitant csDMARDs [24]. There are several contextual factors that contributed to the uniformly low absolute infection rates in this study and may affect generalizability. First, participants were enrolled in low disease activity or remission, a state known to carry lower infection risk than moderate and high disease activity. Second, in the reduction group we excluded baseline glucocorticoids, and overall MTX doses were modest, both of which may attenuate infection risk. Finally, infections were captured using a strict definition in requiring hospitalization by physician-confirmed, and the small number of events leading to discontinuation further reduced observed counts. In addition, this study had shorter disease duration, younger age than the existing registry populations, and lower structural/functional burden (Steinbrocker stage/class and HAQ-DI).

Residual synovitis, often detected via ultrasound in RA patients in clinical remission, predicts flares and structural progression [25]. Ultrasound assessments have been explored to identify patients suitable for tapering. While some studies found no predictive value, others linked shorter untreated disease duration to successful discontinuation [26]. PD scores were associated with tapering failure in both short- and long-term follow-ups [27, 28], and Doppler scores/GS synovial hypertrophy predicted unsuccessful discontinuation [29]. Our analysis identified GS scores before tapering as an independent predictor of successful interval extension and PD scores before discontinuation as predictors of successful bDMARD discontinuation. This aligns with studies linking Doppler findings to successful tapering [30]. However, the lack of consensus on ultrasound remission criteria remains a limitation, influenced by joint selection and ultrasound device sensitivity. Standardized assessment methods are a key priority [30]. Several limitations should be acknowledged. First, this was a single center observational study with a moderate sample size, which may limit generalizability. Although we used propensity score matching and adjusted multivariable models, residual or unmeasured confounding cannot be excluded. Differences in concomitant MTX use across subgroups (e.g., IL-6Ri users) may also act as confounders despite adjustment. While the dose-reduction strategy showed favorable safety—with lower rates of infection and malignancy than standard regimens—and may be particularly beneficial for patients with RA-ILD due to reduced infection risk, vigilance is warranted in patients with residual disease activity or structural damage, who remain at risk of flares and progression; ultrasound evaluation of residual synovitis is crucial in these cases [24]. Additional limitations include the small cohort, the need to clarify long-term effects on joint damage and overall disease control, and the study’s focus on patients in remission for ≥ 6 months, which limits applicability to populations with higher disease activity. Patient-reported outcomes were not collected (e.g., treatment satisfaction, quality of life, pain, fatigue), nor could we assess potential patient–provider perception discrepancies. Future investigations should consider incorporating validated patient-reported outcomes instruments to complement clinical endpoints and better capture the patient’s perspective during tapering and discontinuation. Finally, there are limitations related to MTX exposure and treatment policy. In this study, baseline MTX use was relatively infrequent and the doses were modest, which is likely influenced by our policy that emphasizes early initiation of bDMARD. Consequently, patients enrolled in the tapering group differ from real-world RA cohorts in which moderate-to-high MTX doses are common. Accordingly, our analysis is conditioned on comparatively low MTX exposure, and the impacts of tapering or discontinuation—particularly class-specific flare patterns—may not fully generalize to settings with higher MTX use, and our findings should be interpreted with caution.

We adopted a stepwise taper-then-stop strategy and performed discontinuation—only after patients completed tapering without flare. This approach is intended to reduce infections and other adverse events, improve cost-effectiveness, and respect patient preference, while mitigating risk through gradual dose spacing and close monitoring, including ultrasound assessment of residual synovitis. Although constrained by a single-center design and a moderate sample size, this study was designed to examine the benefits and challenges of bDMARD dose-reduction strategies in routine clinical practice and to inform the establishment of treatment optimization.

After resumption of standard-interval therapy, remission is generally re-attained even among patients who flare; however, caution is warranted regarding the pace of improvement. Many patients experience several months of LDA short of remission before remission is re-established. Taken together, our findings indicate that tapering or discontinuation strategies require a risk–benefit–oriented approach with stringent patient selection, close longitudinal monitoring, and shared decision-making. While acknowledging the limitations of a single center study with a moderate sample size, our tapering strategy supports the reasonableness of attempting drug-free maintenance in a limited subset of patients whose clinical stability is carefully confirmed—including by musculoskeletal ultrasound assessments of residual synovitis.

### Key points in the context of existing studies

Recent studies indicate that, even during sustained remission, patient interest in tapering or discontinuation remains high, yet concerns about flares, infection, and long-term safety persist [31]. While tapering or stopping bDMARDs generally increases flare risk [31–35], the observation that many patients can re-achieve disease control after re-induction [32, 36] is an important consideration when recommending tapering strategies. In our cohort, which was characterized by shorter disease duration and a high proportion of first-line bDMARD users, flare rates during stepwise interval extension (Phase 1) did not differ from standard care, whereas post-discontinuation (Phase 2) was associated with a significant increase in flares. Predictor analyses underscore the importance of identifying candidates who can taper safely. In our data, residual synovitis on musculoskeletal ultrasound, GS score y during interval extension, and, after discontinuation, MTX non-use, ACPA positivity, and PD score were each associated with higher flare risk. Notably, MTX non-use independently predicted post-discontinuation flare (odds ratio 3.69, 95% CI 2.89–4.26). By contrast, in Hashimoto et al. (37) MTX non-use was not an independent predictor. This discrepancy may reflect differences in bDMARD class composition and patient characteristics. Relative to Hashimoto et al., our cohort included fewer TNFi users (43.6% vs. 70.2%), more IL-6Ri users (50.0% vs. 20.4%), and shorter disease duration. Consistent with Nishimoto et al. [23], MTX-free discontinuation of the IL-6Ri tocilizumab yielded a 52-week low-disease-activity maintenance rate of 13.4% (95% CI 8.4–18.3), whereas in our study IL-6Ri with concomitant MTX achieved a 52-week non-flare rate of 20.9% (data not shown).

Immunologic and imaging markers supported these patterns. Post-discontinuation flares were associated with ACPA positivity rather than RF (38), and residual synovitis, particularly PD, predicted flares during tapering (39). In our cohort, lower baseline GS at the start of interval extension and lower PD at the start of discontinuation were each associated with fewer flares, and both GS and PD remained independent after covariate adjustment. Finally, the Reduction group experienced fewer AEs leading to treatment discontinuation than the control group, which suggests potential safety benefits, including reduced infection risk, and economic advantages in addition to decreased drug exposure.

## Conclusion

Our results demonstrate the benefits and challenges of bDMARD dose-reduction strategies in routine clinical practice for appropriately selected patients. Future research should focus on refining flare-risk prediction models, incorporating comprehensive evaluation metrics that include patients’ quality of life and treatment satisfaction, assessing the contribution of bDMARD dose reduction strategies to healthcare economics, and evaluating the long-term effects of dose reduction to establish evidence-based treatment optimization for RA.

## Supplementary Information

Supplementary Material 1.

Supplementary Material 2.

Supplementary Material 3.

Supplementary Material 4.

Supplementary Material 5.

Supplementary Material 6.

## Data Availability

All data relevant to the study are included in the article.

## Abbreviations

ACPA: Anti-citrullinated peptide antibody
ACR: American college of rheumatology
AE: Adverse event
BUC: Bucillamine
bDMARD: Biological disease-modifying antirheumatic drug
CI: Confidence interval
CRP: C-reactive protein
csDMARD: Conventional synthetic disease-modifying antirheumatic drug
CTLA4-Ig: Cytotoxic t-lymphocyte antigen 4 immunoglobulin
DAS28: Disease activity score in 28 joints
DrVAS: Physician visual analogue scale
ESR: Erythrocyte sedimentation rate
EULAR: European league against rheumatism
GS: Gray scale (ultrasound)
HAQ-DI: Health assessment questionnaire disability index
HR: Hazard ratio
IGU: Iguratimod
IL-6Ri: Interleukin-6 receptor inhibitor
IQR: Interquartile range
LDA: Low disease activity
MMP-3: Matrix metalloproteinase-3
MTX: Methotrexate
OMERACT: Outcome measures in rheumatology
OR: Odds ratio
PD: Power doppler (ultrasound)
PS: Propensity score
PtVAS: Patient visual analogue scale
RA: Rheumatoid arthritis
RF: Rheumatoid factor
SAE: Serious adverse event
SASP: Sulfasalazine
SD: Standard deviation
TAC: Tacrolimus
TNFi: Tumor necrosis factor inhibitor
tsDMARD: Targeted synthetic disease-modifying antirheumatic drug
VAS: Visual analogue scale
