# Supplementary figures and images for: Stepwise dose reduction and discontinuation of bDMARD in rheumatoid arthritis: a prospective cohort study of flare-free population, flares, and predictive markers
Source: Arthritis Res Ther. 2025 Nov 4;27:205. doi: 10.1186/s13075-025-03672-y (PMC12584375; doi:10.1186/s13075-025-03672-y)

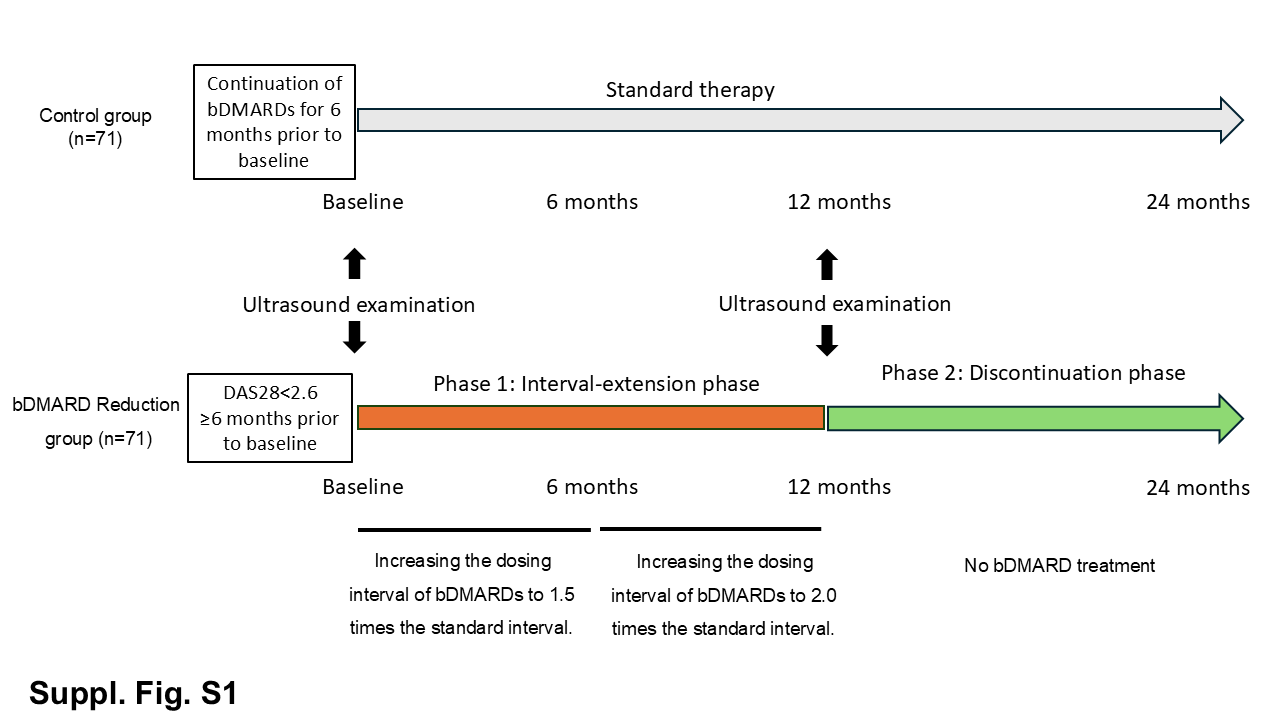

Supplement: Supplementary file 1 — Supplementary Material 1. [file 13075_2025_3672_MOESM1_ESM.tif]

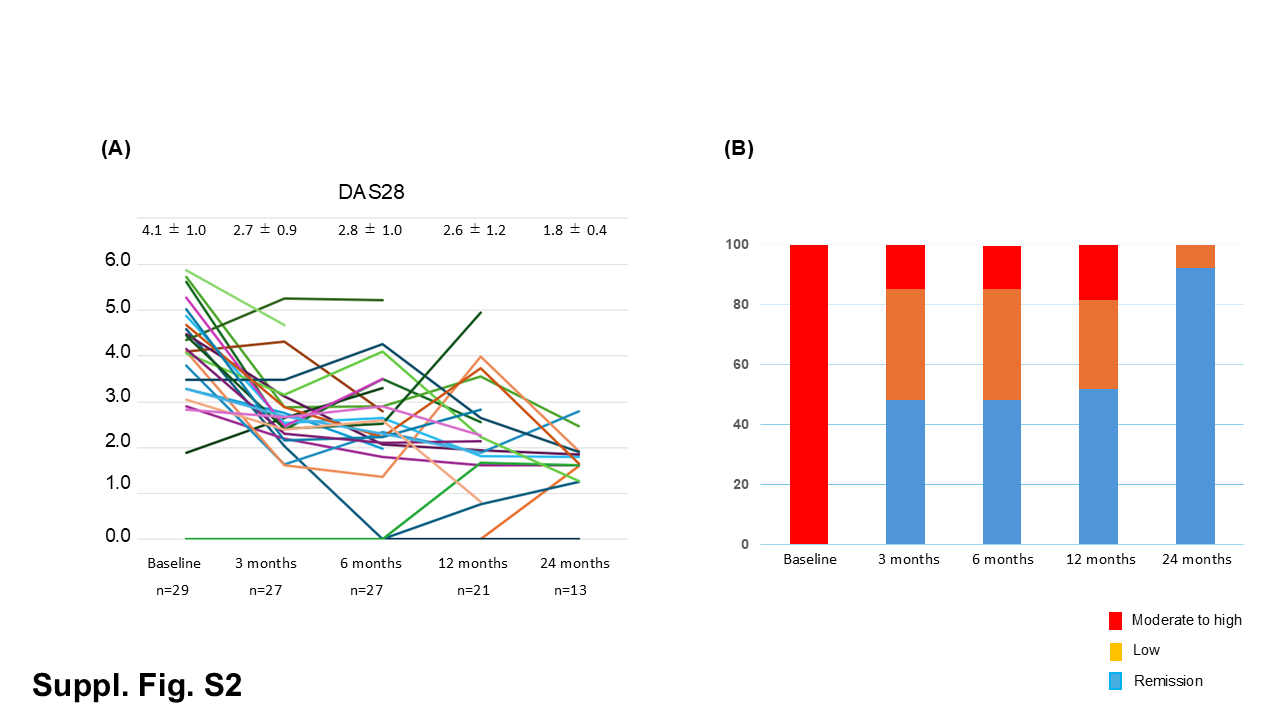

Supplement: Supplementary file 2 — Supplementary Material 2. [file 13075_2025_3672_MOESM2_ESM.tif]

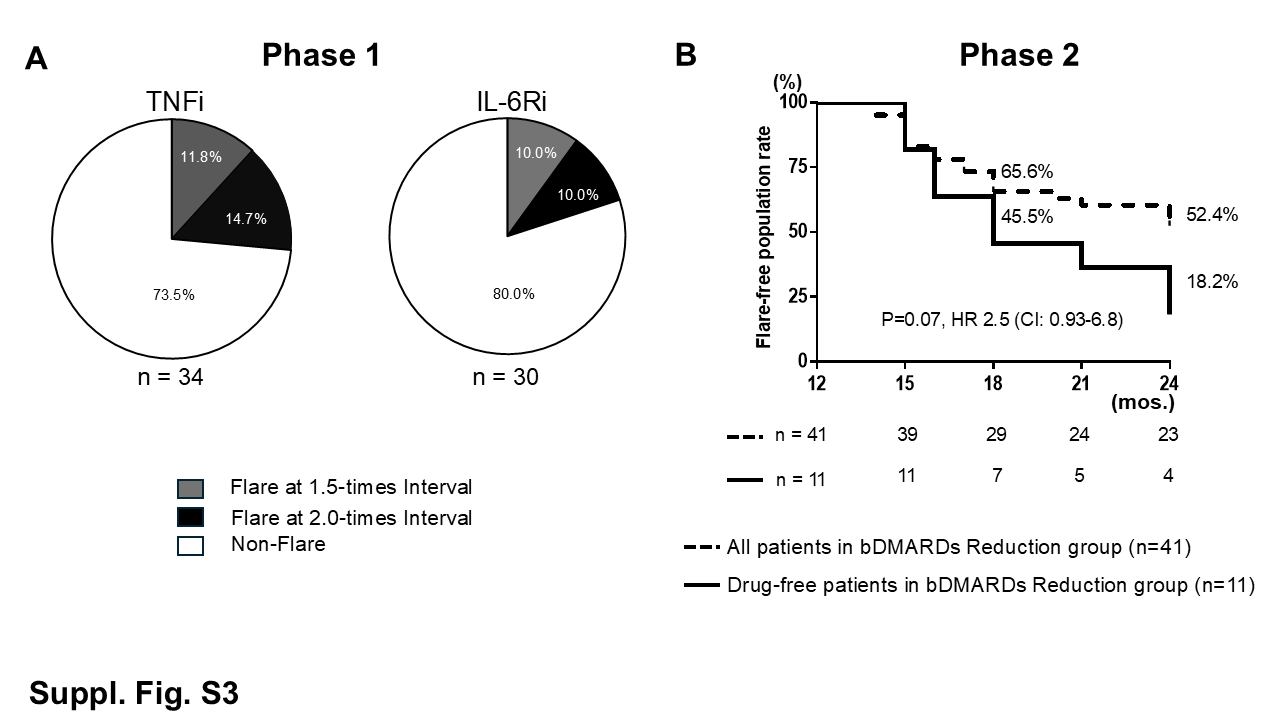

Supplement: Supplementary file 3 — Supplementary Material 3. [file 13075_2025_3672_MOESM3_ESM.tif]
