# Supplementary material for: Stepwise dose reduction and discontinuation of bDMARD in rheumatoid arthritis: a prospective cohort study of flare-free population, flares, and predictive markers
Source: Arthritis Res Ther. 2025 Nov 4;27:205. doi: 10.1186/s13075-025-03672-y (PMC12584375; doi:10.1186/s13075-025-03672-y)
Supplement: Supplementary file 4 — Supplementary Material 4. [file 13075_2025_3672_MOESM4_ESM.docx]

**Supplementary Table S1.** Bilateral synovial intra-articular recesses and periarticular sites evaluated by ultrasound in both joints

| **Joint** | **Anatomical area(s)** |
| --- | --- |
| MCP (1–5), PIP (1–5) | Dorsal recess |
| Wrist | Radiocarpal recesses |
|  | Ulnarcarpal recesses |
|  | Intercarpal recesses |
| Elbow | Humeroradial joint |
|  | Humeroulnar joint |
|  | Posterior recesses |
| Shoulder | Subdeltoid bursa |
|  | Biceps sheath |
|  | Subacromial bursa |
| Knee | Suprapatellar recesses |
|  | Medial parapatellar recess |
|  | Lateral parapatellar recess |
|  | Medial femorotibial joint line |
|  | Lateral femorotibial joint line |

The data were obtained in the the ultrasound (US) examination of synovial hypertrophy and vascularization and evaluated using gray-scale imaging and power Doppler (PD) across 48 sites in 28 joints. The gray scale and PD results are scored qualitatively for each joint on a scale of 0–3, with both having a total score range of 0–144. MCP: metacarpophalangeal joint, PIP: proximal interphalangeal joint.
