# Supplementary material for: Stepwise dose reduction and discontinuation of bDMARD in rheumatoid arthritis: a prospective cohort study of flare-free population, flares, and predictive markers
Source: Arthritis Res Ther. 2025 Nov 4;27:205. doi: 10.1186/s13075-025-03672-y (PMC12584375; doi:10.1186/s13075-025-03672-y)
Supplement: Supplementary file 5 — Supplementary Material 5. [file 13075_2025_3672_MOESM5_ESM.docx]

**Supplementary Table S2. Phase 2 flare after bDMARD discontinuation stratified by MTX use within each biologic class**

| **MOA** | **bDMARDs** | **n** | **Flare, n (%)** | **Time to flare after discontinuation, months** | **Flare rates with concomitant MTX** | |
| --- | --- | --- | --- | --- | --- | --- |
|  |  |  |  |  | **MTX use, n (%)** | **MTX non-use, n (%)** |
| Classes | TNFi | 21 | 7 (33.3) | 11.7 ± 12.5 | 3/12 (25.0) | 4/9 (44.4) |
|  | IL-6Ri | 17 | 9 (52.9) | 6.4 ± 3.9 | 3/8 (37.5) | 6/9 (66.7) |
|  | CTLA4-Ig | 3 | 1 (33.3) | 2.5 ± 0.7 | 1/2 (50.0) | 0/1 (0.0) |
|  | Infliximab | 4 | 0 (0.0) | 0.0 | ― | ― |
|  | Adalimumab | 5 | 2 (40.0) | 14.0 ± 15.6 | ― | ― |
|  | Etanercept | 4 | 2 (50.0) | 5.5 ± 4.9 | ― | ― |
|  | Certolizumab | 8 | 3 (37.5) | 12.5 ± 16.4 | ― | ― |
|  | Tocilizumab | 9 | 5 (44.4) | 7.5 ± 3.4 | ― | ― |
|  | Sarilumab | 8 | 4 (50.0) | 5.3 ± 4.5 | ― | ― |
|  | Abatacept | 3 | 1 (33.3) | 2.5 ± 0.7 | ― | ― |

Classes definitions: TNFi (adalimumab, etanercept, infliximab, certolizumab pegol), IL-6Ri (tocilizumab, sarilumab), and CTLA4-Ig (abatacept). MOA: mode of action, bDMARDs: biological disease-modifying antirheumatic drugs, TNFi: tumor necrosis factor inhibitor, IL-6Ri: inteleukin-6 receptor inhibitor, CTLA4-Ig: cytotoxic T-lymphocyte antigen 4 immunoglobulin, MTX: methotrexate
