# Supplementary material for: Stepwise dose reduction and discontinuation of bDMARD in rheumatoid arthritis: a prospective cohort study of flare-free population, flares, and predictive markers
Source: Arthritis Res Ther. 2025 Nov 4;27:205. doi: 10.1186/s13075-025-03672-y (PMC12584375; doi:10.1186/s13075-025-03672-y)
Supplement: Supplementary file 6 — Supplementary Material 6. [file 13075_2025_3672_MOESM6_ESM.docx]

**Supplementary Table S3. Baseline characteristics and standardized mean differences: withdrawal-censored versus observed**

| **Variable** | **Type** | **A: n** | **A: mean** | **A: SD** | **B: n** | **B: mean** | **B: SD** | **Absolute SMD** | **A: proportion** | **B: proportion** |
| --- | --- | --- | --- | --- | --- | --- | --- | --- | --- | --- |
| Age (years) | continuous | 116.00 | 60.23 | 17.26 | 253.00 | 61.77 | 15.17 | 0.10 |  |  |
| Disease duration (months) | continuous | 115.00 | 82.37 | 88.69 | 253.00 | 79.65 | 92.22 | 0.03 |  |  |
| HAQ-DI (baseline) | continuous | 89.00 | 0.65 | 0.74 | 185.00 | 0.55 | 0.84 | 0.13 |  |  |
| CRP (mg/dL, baseline) | continuous | 116.00 | 0.57 | 1.20 | 254.00 | 0.30 | 0.67 | 0.31 |  |  |
| GS score (ultrasound, baseline) | continuous | 81.00 | 16.28 | 16.00 | 157.00 | 14.82 | 12.76 | 0.10 |  |  |
| PD score (ultrasound, baseline) | continuous | 70.00 | 5.51 | 8.18 | 152.00 | 2.70 | 4.85 | 0.46 |  |  |
| Female (%) | binary | 116.00 |  |  | 254.00 |  |  | 0.07 | 0.76 | 0.79 |
| ACPA positive (%) | binary | 116.00 |  |  | 254.00 |  |  | 0.05 | 0.83 | 0.81 |
| RF positive (%) | binary | 26.00 |  |  | 59.00 |  |  | 0.01 | 0.79 | 0.76 |
| MTX use (%) | binary | 116.00 |  |  | 254.00 |  |  | 0.07 | 0.55 | 0.59 |

*A (Withdrawal-censored)*: patients who discontinued prior to flare at any step of tapering (Phase 1 or 2); observations were right-censored at the withdrawal date with no subsequent follow-up analyzed. *B (Observed)*: patients who remained under observation and either experienced a flare or were administratively censored at study end. For primary time-to-event outcomes, censoring ≠ missing; thus no missingness arose.
SMD: continuous variables (including GS and PD numeric scores) use ∣Δmean∣/SDpooled​; binary variables use ∣p1−p2∣/√p(1-p) with the pooled proportion. Absolute SMD <0.10 = excellent balance; <0.15 = acceptable. CRP and power Doppler exceeded 0.15 and were adjusted for in multivariable models. Units/coding: CRP mg/L; disease duration months; GS and PD are recorded as numeric semiquantitative scores (e.g., 0–3 per joint with sums or means across assessed joints, as specified in Methods). HAQ-DI: health assessment questionnaire–disability index, CRP: C-reactive protein, GS: Gray-scale, PD: Power Doppler, ACPA: anti-citrullinated peptide antibody, RF: rheumatoid factor, MTX: methotrexate, SD: standard deviation, SMD: standardized mean difference, p: pooled proportion
